# Supplementary figures and images for: Single-cell transcriptomics unveils skin cell specific antifungal immune responses and IL-1Ra- IL-1R immune evasion strategies of emerging fungal pathogen Candida auris
Source: PLoS Pathog. 2024 Nov 13;20(11):e1012699. doi: 10.1371/journal.ppat.1012699 (PMC11588283; doi:10.1371/journal.ppat.1012699)

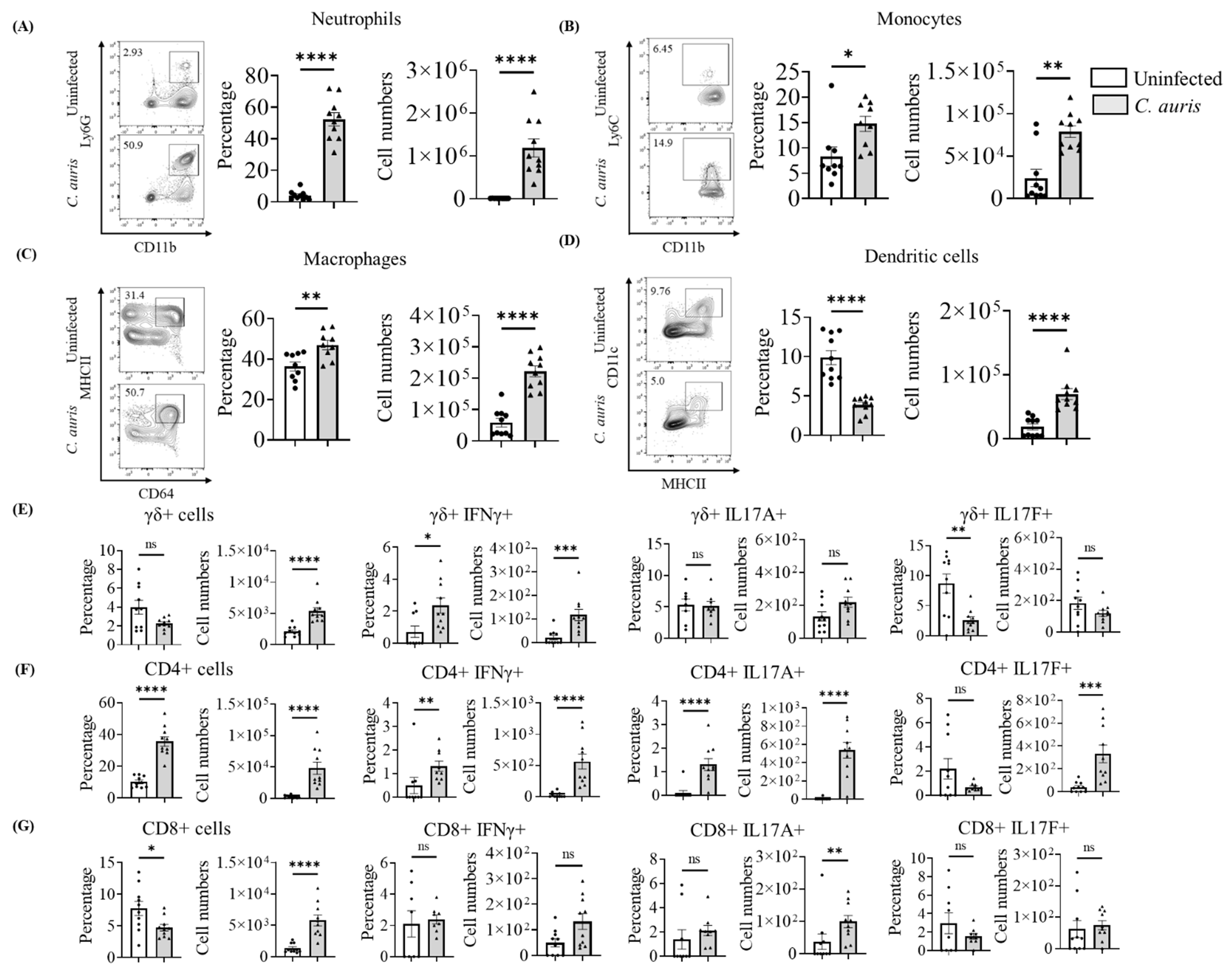

Supplement: S1 Fig — The representative flow plot and the bar graph represent the percentage and absolute number of (A) neutrophils, (B) Monocytes, (C) Macrophages, and (D) dendritic cells in the C. auris infected group compared to the uninfected group. The bar graph represents the percentage and absolute number of (E) γδ+ cells, γδ+ IFNγ+ cells, γδ+ IL17A+ cells, and γδ+ IL17F+ cells, (F) CD4+ cells, CD4+ IFNγ+ cells, CD4+ IL17A+ cells, CD4+ and IL17F+ cells, (G) CD8+ cells, CD8+ IFNγ+ cells, CD8+ IL17A+ cells, CD8+ and IL17F+ cells. Twelve mice were used from each group, and the error bar represents the mean ± SEM. * p < 0.05, ** p <0.01, *** p <0.001, **** p <0.0001. (TIF) [file ppat.1012699.s001.tif]

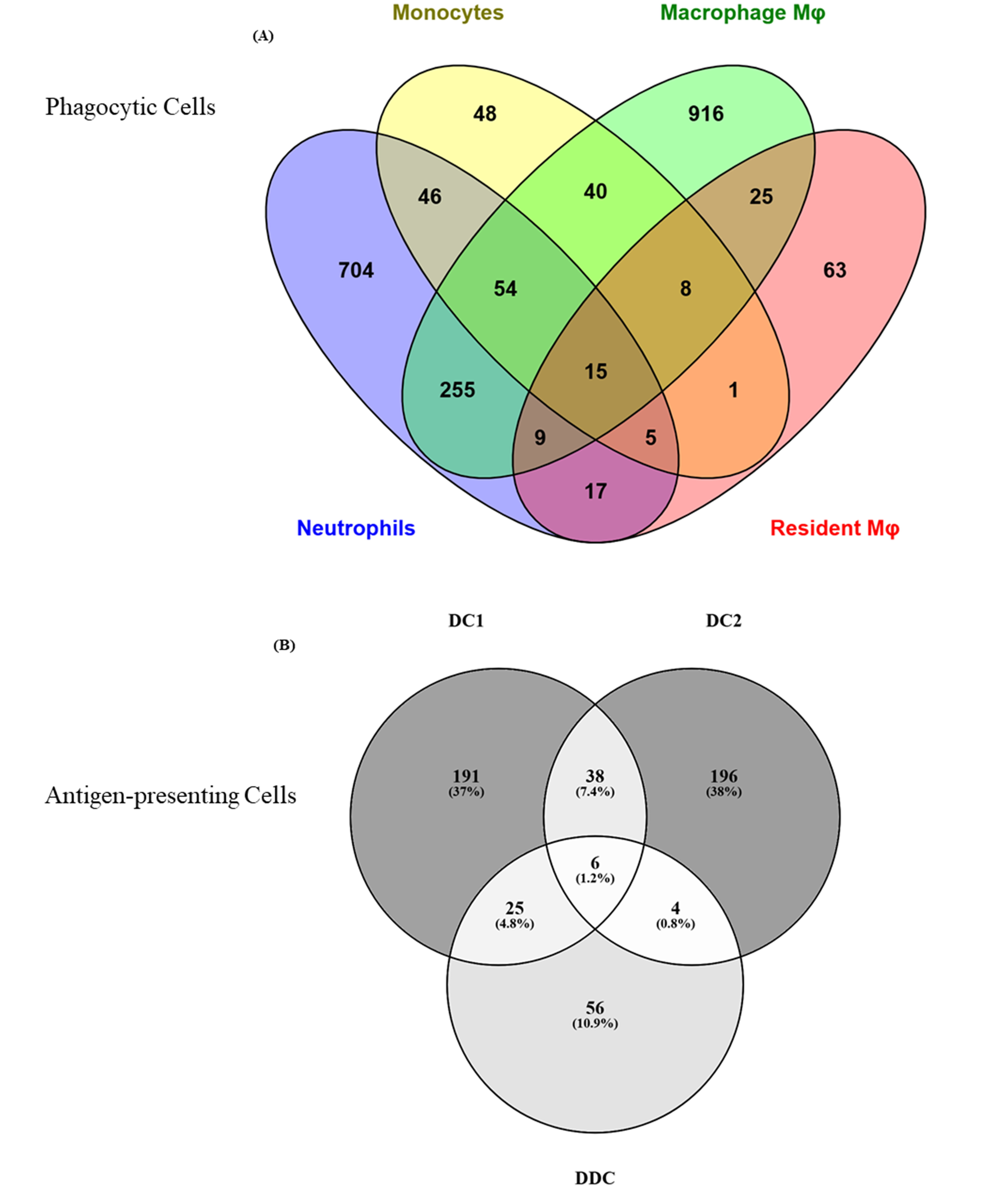

Supplement: S2 Fig — Venn diagram showing the number of upregulated genes shared within the (A) phagocytic cells and (B) antigen presenting cells upon C. auris infection. (TIF) [file ppat.1012699.s002.tif]

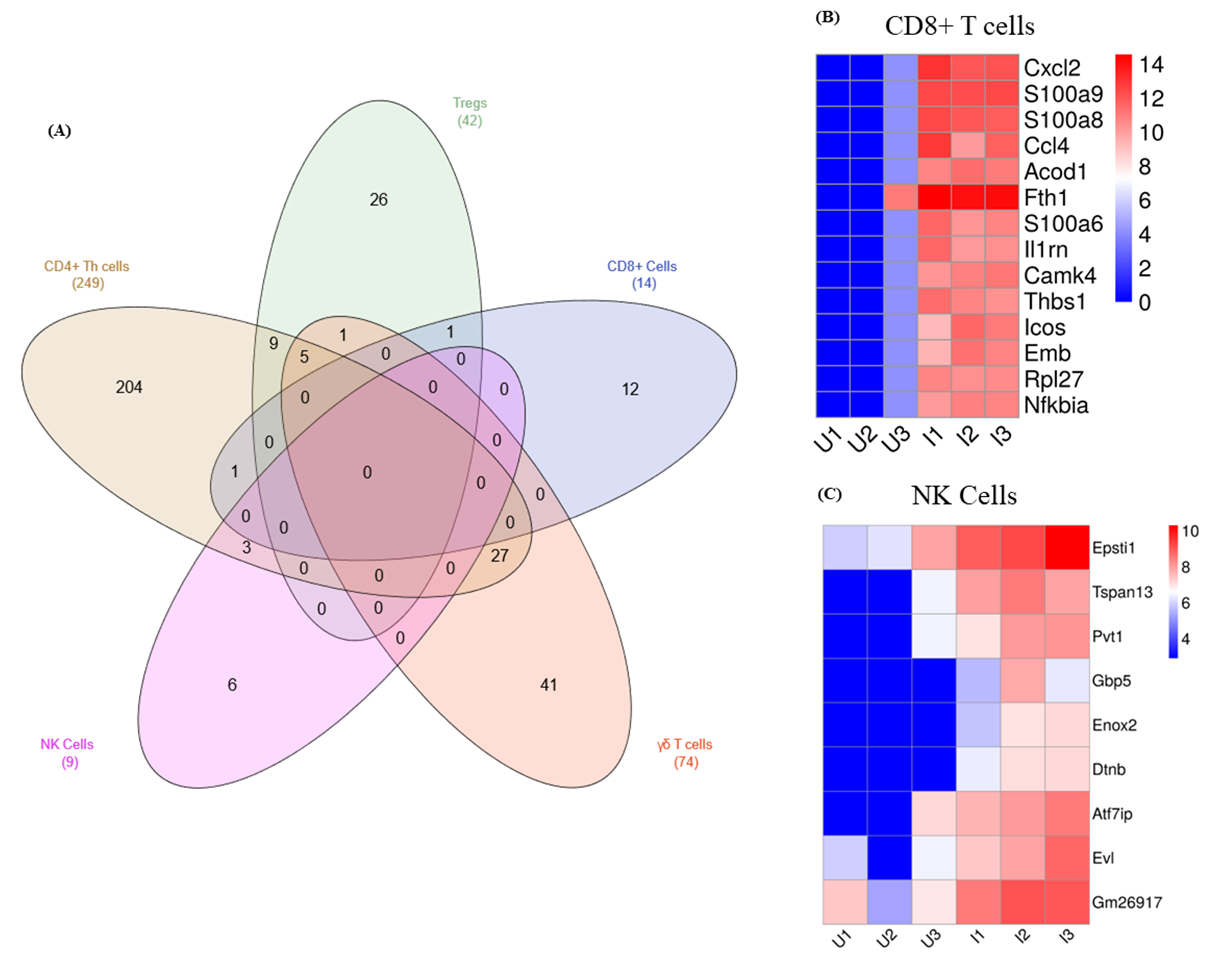

Supplement: S3 Fig — (A) Venn diagram showing the number of upregulated genes shared within lymphoid subsets. The heatmap represents the expression of the significantly upregulated genes in (B) CD8+ and (C) NK cells in uninfected and infected groups. Upregulated genes with Log 2-fold change + 2 and FDR > 5% were represented. The normalized gene counts were plotted in the heatmap, and the scale indicates red for high, blue for low, and white for moderate expression in the samples. Each column represents a different sample. (TIF) [file ppat.1012699.s003.tif]

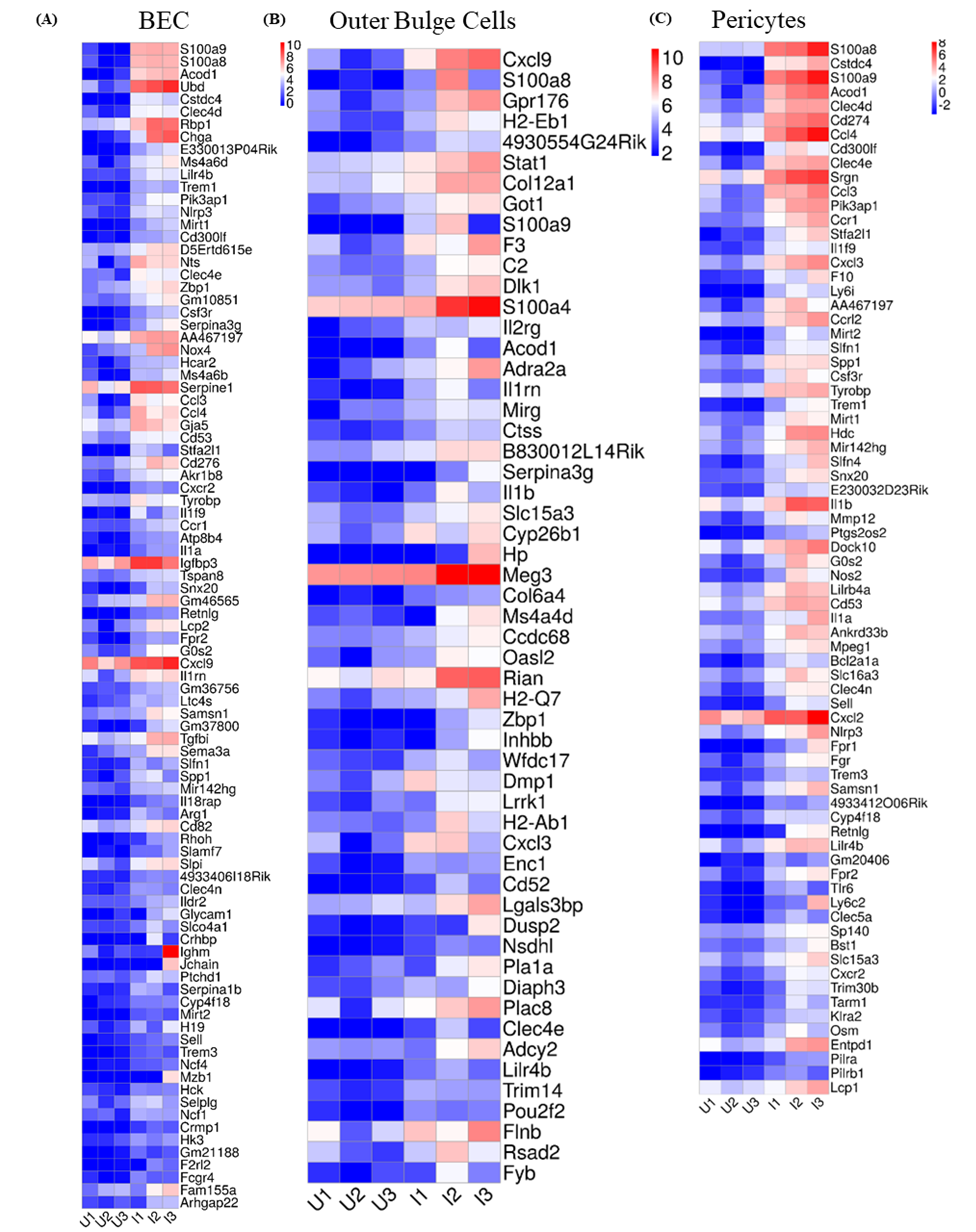

Supplement: S4 Fig — The heatmap represents the expression of the significantly upregulated genes in (A) BEC (B) outer bulge cells and (C) pericytes cells in uninfected and infected groups. Upregulated genes with Log 2-fold change ≥ 2 and FDR > 5% were represented. The normalized gene counts were plotted in the heatmap, and the scale indicates red for high, blue for low, and white for moderate expression in the samples. Each column represents a different sample. (TIF) [file ppat.1012699.s004.tif]

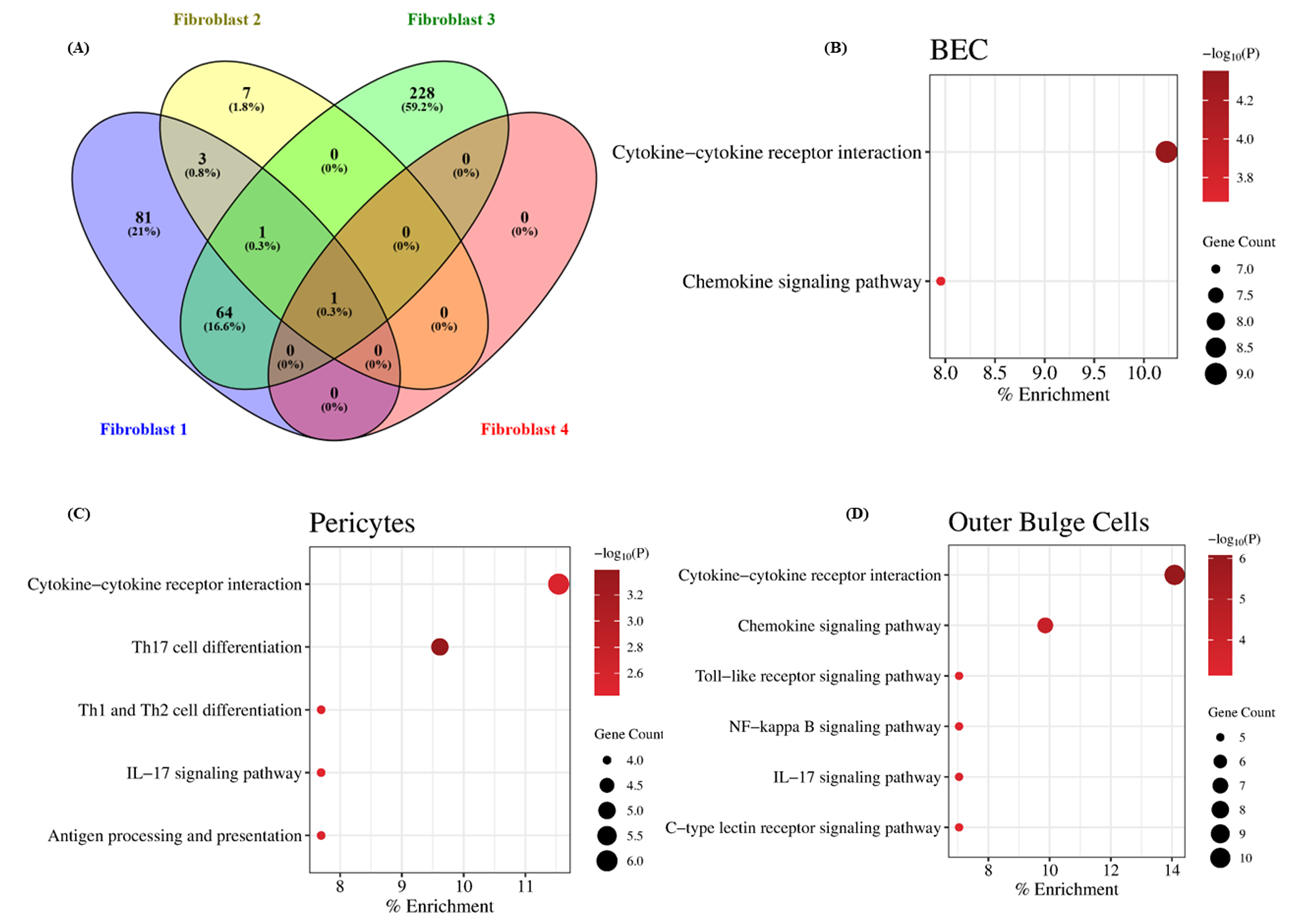

Supplement: S5 Fig — (A) Venn diagram showing the number of significantly upregulated genes shared within the identified fibroblast subsets. The bubble plot represents the KEGG pathways of the enriched upregulated genes of the (B) BEC, (C) pericytes and (D) outer bulge cells. The X-axis denotes the percentage enrichment of the KEGG pathways. (TIF) [file ppat.1012699.s005.tif]

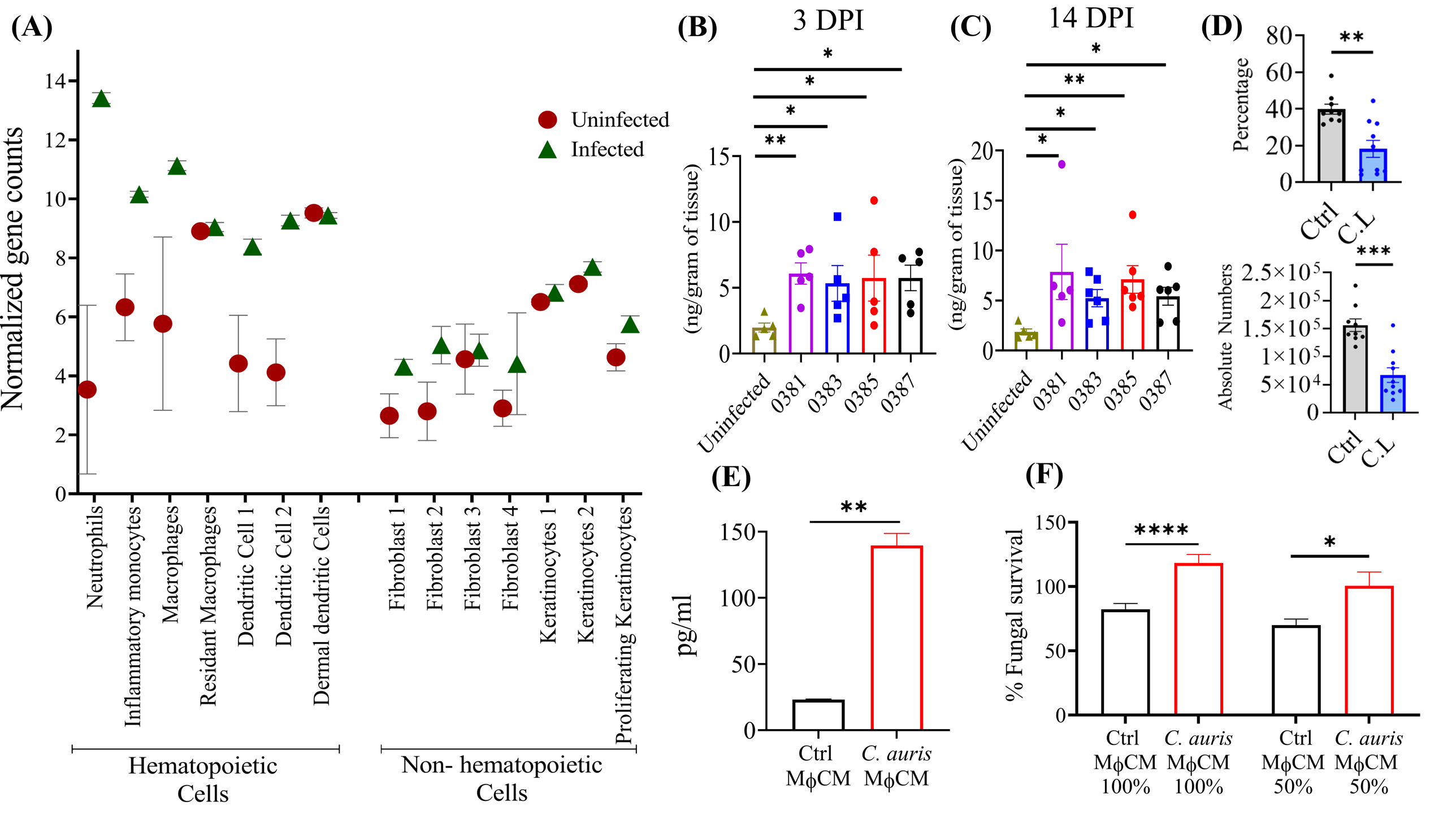

Supplement: S6 Fig — (A) The normalized read counts of Il1rn gene in the hematopoietic and non-hematopoietic cell types from the scRNA-seq dataset. Both uninfected and infected samples were plotted and the mean were represented with the error bar. The IL-1Ra level in skin tissues of mice groups received PBS, Candida auris South Asian clade AR0387, East Asian clade AR0381, African clade AR0383, or South American clade AR0385 after day. (B) 3 p.i. and (C) 14 p.i. (n = 5–7 mice/group). (D) Percentage and absolute number of F4/80+ MHCII+ macrophage in the infected skin tissue of mice injected with clodrosome (C.L) or 1X PBS (Ctrl) injected mice groups. (E) Measurement IL-1Ra levels from the culture supernatant of BMDM alone or BMDM stimulated with C. auris 0387 for 16 h. (n = 12). Error bars represent mean ± SEM. ** p <0.01. (F) The bar graph represents the fungal survival of C. auris 0387 primed with neutrophils in the presence of MΦ CM collected from BMDM alone (Ctrl MΦ CM) or BMDM stimulated with C. auris 0387 (C. auris MΦ CM) for 16 h (n = 16 to 19). 50% CM was diluted with complete DMEM. Error bars represent mean ± SEM. * p <0.05, **** p <0.0001. Abbreviations—BMDM, bone marrow-derived macrophages; MΦ CM, macrophage conditioned medium. Statistical significances were calculated using Mann–Whitney U. (TIF) [file ppat.1012699.s006.tif]

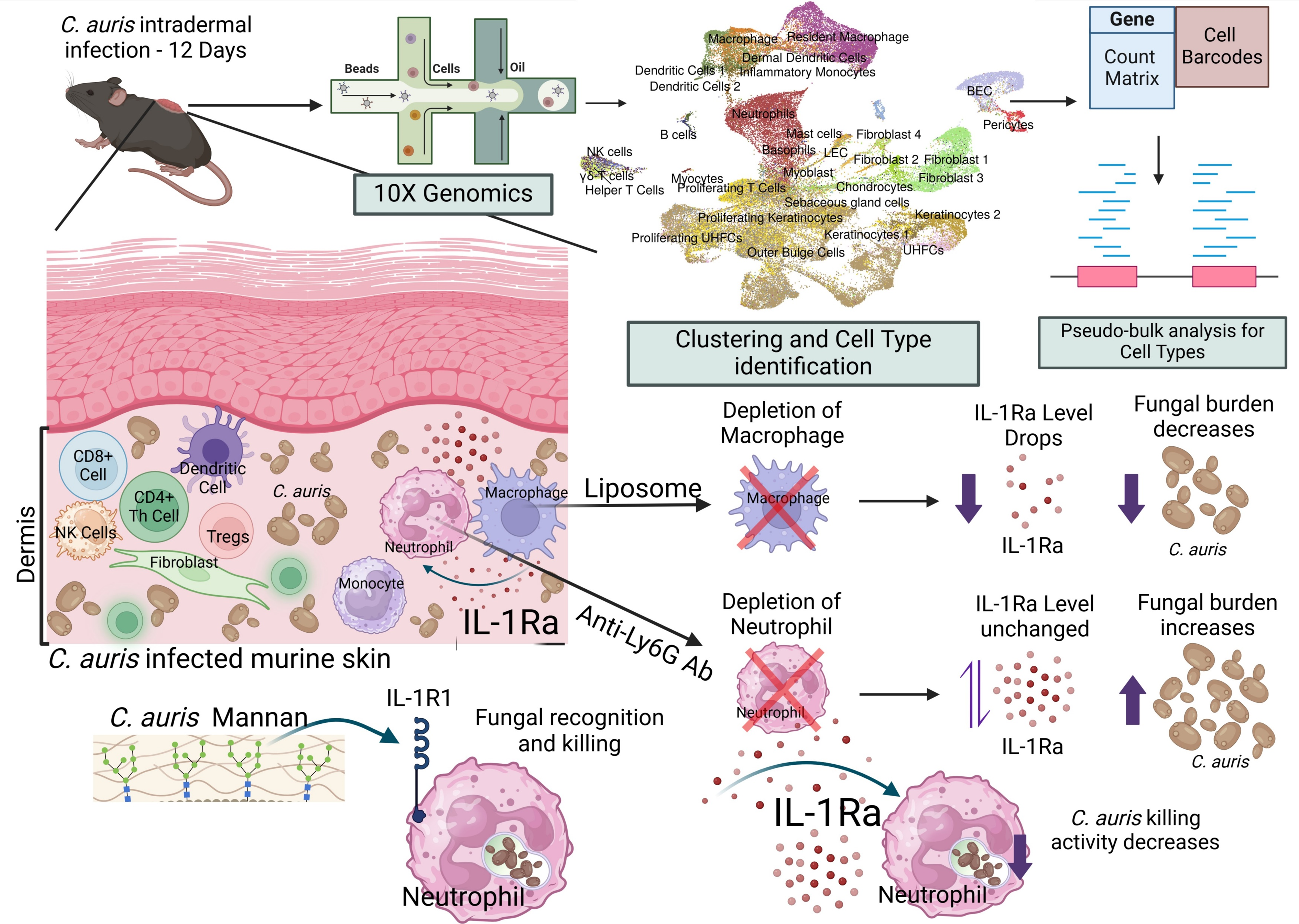

Supplement: S7 Fig — (TIF) [file ppat.1012699.s007.tif]
