# Supplementary material for: Single-cell transcriptomics unveils skin cell specific antifungal immune responses and IL-1Ra- IL-1R immune evasion strategies of emerging fungal pathogen Candida auris
Source: PLoS Pathog. 2024 Nov 13;20(11):e1012699. doi: 10.1371/journal.ppat.1012699 (PMC11588283; doi:10.1371/journal.ppat.1012699)
Supplement: S1 Table — The mean cell numbers of the cell type in the uninfected and infected samples were represented. The fold changes in the cell types recruited in the infected samples compared to the uninfected control were represented. (DOCX) [file ppat.1012699.s008.docx]

**Table S1:** The number of cells in each cell type identified in the infected and uninfected groups. The mean cell numbers of the cell type in the uninfected and infected samples were represented. The fold changes in the cell types recruited in the infected samples compared to the uninfected control were represented.
