# Supplementary material for: Single-cell transcriptomics unveils skin cell specific antifungal immune responses and IL-1Ra- IL-1R immune evasion strategies of emerging fungal pathogen Candida auris
Source: PLoS Pathog. 2024 Nov 13;20(11):e1012699. doi: 10.1371/journal.ppat.1012699 (PMC11588283; doi:10.1371/journal.ppat.1012699)
Supplement: S2 Table — The mean UMIs in uninfected and infected sample are represented in the table. (DOCX) [file ppat.1012699.s009.docx]

**Table S2:** The total UMIs and annotated genes identified in the infected and uninfected groups. The mean UMIs in uninfected and infected sample are represented in the table.

**
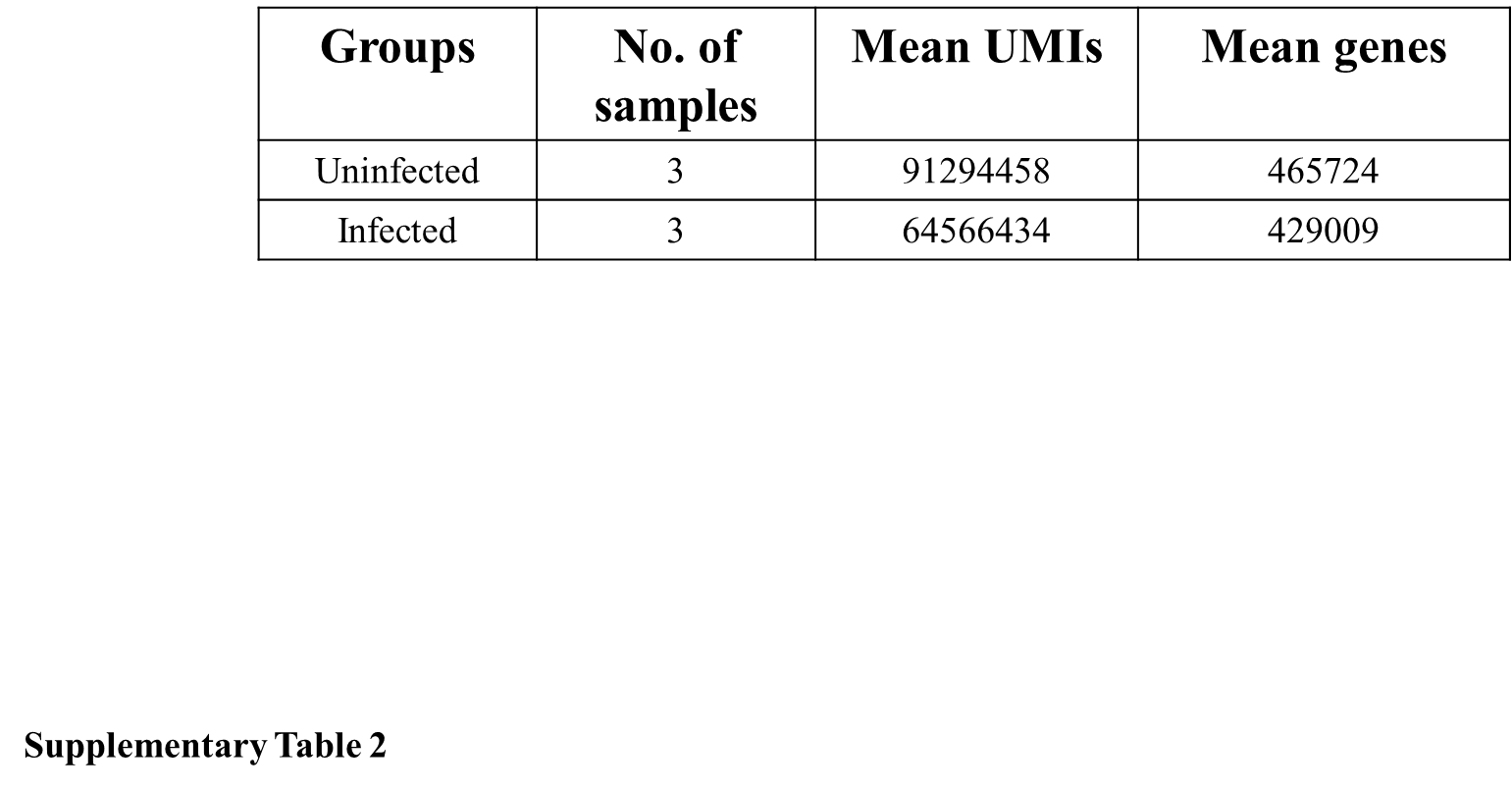
**
