# Supplementary material for: Single-cell transcriptomics unveils skin cell specific antifungal immune responses and IL-1Ra- IL-1R immune evasion strategies of emerging fungal pathogen Candida auris
Source: PLoS Pathog. 2024 Nov 13;20(11):e1012699. doi: 10.1371/journal.ppat.1012699 (PMC11588283; doi:10.1371/journal.ppat.1012699)
Supplement: S3 Table — (DOCX) [file ppat.1012699.s010.docx]

**Table S3:** The DEGs of myeloid subsets enriched in the KEGG pathways upon *C. auris* murine skin infection.

| **KEGG Pathways** | **DEGs enriched in the pathway** |
| --- | --- |
| Phagosome | **Neutrophils** - *Ctss, Sec61g, H2-T23, Canx, Itgav, Fcgr3, Atp6v0d2, Atp6v1c1, Atp6v1b2, H2-Q10, Ncf1, Atp6v1g1, Itgb1, Cd14, H2-K1, Atp6v0c, Rab7, Atp6v1a, H2-Q6, M6pr, Atp6v1d, Tap2, Olr1, Rac1, Tap1, Lamp2, Tubb6, Lamp1, Tlr6, Tlr4, Stx7, Tlr2, C3, H2-Q7, H2-T22, H2-Aa, H2-Ab1, Cybb, Atp6v1e1, Atp6v0d1, Atp6v0a1, H2-D1, Eea1* and *Atp6v0e*  **Inflammatory Monocytes** - *Fcgr2b, H2-Eb1, Mrc1, Tap1, Tlr2* and *Tlr6*  **Macrophage** – *Ctss, Rab5c, Fcgr2b, H2-Q6, Sec22b, Dync1li1, Olr1, Itgav, Tubb5, Thbs1, Tuba1c, Tubb4b, Tlr2, C3, Cd14* and *Ctsl*  **Resident Macrophage** – *Marco, Olr1, Fcgr4, C3* and *Thbs1*  **Dendritic cell 1** – *Cd209a, Tap1, Olr1, Tlr2, Fcgr3, H2-DMb2, Fcgr4, Cybb* and *Fcgr1*  **Dendritic cell 2** – *H2-Q6, Tap1, H2-DMb2, Stx7, H2-Eb1, Atp6v0b, H2-Q7, H2-Aa, Cybb, H2-Ab1, Atp6v1g1, H2-DMa,* and *Coro1a*  **Dermal Dendritic cells** – *Msr1, Fcgr4, C3* and *Fcgr1* |
| Endocytosis | **Neutrophils** -*Cltc, Mdm2, Pdcd6ip, Rab11fip1, Chmp2a, Traf6, Smap1, Wipf1, Clta, Arfgef1, H2-K1, Smad3, Rab7, Rab11a, Arrb1, Rab31, Igf2r, Wipf2, Igf1r, Eps15, H2-T22, Hspa1b, Arpc4, Hspa8, Washc4, H2-T23, Ehd1, Kif5b, Vps4b, Hspa1a, Arf5, H2-Q10, Arf4, Arap1, Rab22a, Arpc2, Il2rg, H2-Q6, Arpc5, Psd4, Washc2, Tgfbr1, Stam2, H2-Q7, Cdc42, Capza2, Iqsec1, Eea1,* and *H2-D1*  **Inflammatory Monocytes** - *Dab2, Bin1, Igf1r, Ccr5, Arrb2,* and *Iqsec1*  **Macrophage** – *Itch, Rab5c, Cav2, Smurf1, Smurf2, Traf6, Wipf1, Src, Hspa1a, Snf8, Vps37b, Washc5, Snx12, Arfgef1, Chmp1a, Ldlrap1, Chmp1b, H2-Q6, Arrb1, Pip5k1a, Dab2, Capza1, Bin1, Dnm2, Igf1r, Snx1, Nedd4l, Stam2, Vps37a, Cav1, Gbf1, Agap1, Chmp5* and *Epn1*  **Resident Macrophage** – *Il2ra*  **Dendritic cell 1** – *Il2rg, Ccr5, Pip5k1c,* and *Cxcr4*  **Dendritic cell 2** – *Hspa1a, H2-Q6, Snx3, Rab8a, H2-Q7, Wipf1, Grk5, Capza2* and *Nedd4l*  **Dermal Dendritic cells** – *Cxcr2* |
| Efferocytosis | **Neutrophils** - *Hif1a, Sirpb1b, Rac1, Cd24a, Adam17, Itgav, Mapkapk2, Dusp16, Jak2, Sirpa, Tgfb1, Slc2a1, Ptpn6, Elmo1, Map2k1, Cebpb, Cd47, Tmem30a, Bsg, Pecam1, Rab7,* and *Lipa*  **Inflammatory Monocytes** - *Ptgs2, Hif1a, C1qb, Arg1, Cd24a,* and *Slc2a1*  **Macrophage** – *Ptgs2, Ptpn11, Mertk, Rab5c, Lrp1, Itgav, Thbs1, Mapk1, Mapk3, Arg2, Map2k1, Arg1, Sgk1, Arnt, Nfatc1, Bsg, Pecam1, Abca1, Ppard, Hif1a, Rab14, Cd24a, Slc2a1, Vps8, Atp11b, Sirpb1c,* and *Havcr2*  **Resident Macrophage** – *Arg2, Sirpb1b, Arg1, Sirpb1c, Sirpb1a, Thbs1, Slc2a1, Nr1h3, Sirpd,* and *Havcr2*  **Dendritic cell 1** – *Ptgs2, Hif1a, Arg1, Atp2a1, Adam17, Sirpb1c,* and *Slc2a1*  **Dendritic cell 2** – *Ptgs2*, *Hif1a, Arg2, Arg1*, and *Mapk3*  **Dermal Dendritic cells** – *Arg1, Sirpb1c,* and *Sirpd* |
| Ubiquitin mediated proteolysis | **Neutrophils** - *Wwp2, Herc4, Mdm2, Ube2a, Ube2g1, Fbxw11, Ube2n, Traf6, Cul3, Ube2d2a, Cul1, Ube2s, Ubc, Socs3, Ube2j1, Socs1, Ube2i, Birc6, Ube2z, Birc3, Herc1,* and *Xiap*  **Inflammatory Monocytes** - *Wwp2,* and *Socs1*  **Macrophage** – *Cul2, Itch, Wwp2, Herc4, Ube2g1, Ube2f, Smurf1, Smurf2, Ube2b, Traf6, Cul3, Cul4a, Ube3a, Ube2k, Cop1, Ube2l3, Cul5, Anapc16, Uba6, Map3k1, Uba1, Nedd4l, Pias1, Socs3,* and *Birc2*  **Dendritic cell 1** – *Ube2l6,* and *Anapc2*  **Dendritic cell 2** – *Nedd4l,* and *Socs3* |
| Fc gamma R-mediated phagocytosis | **Neutrophils** - *Arpc2, Prkcd, Pik3cb, Arpc5, Hck, Marcksl1, Rac1, Fcgr3, Inpp5d, Lyn, Vav1, Syk, Map2k1, Pik3r1, Vav3, Ncf1, Pik3ca, Cdc42, Arpc4,* and *Marcks*  **Inflammatory Monocytes** - *Fcgr2b, Pik3cb, Marcksl1, Bin1,* and *Pla2g4a*  **Macrophage** – *Fcgr2b, Pip5k1a, Pik3cb, Map2k1, Pik3r1, Bin1, Dnm2, Rac2, Pla2g4a, Was, Mapk1,* and *Mapk3*  **Resident Macrophage** – *Fcgr4*  **Dendritic cell 1** – *Ptprc, Fcgr3, Fcgr4, Rac2, Pip5k1c, Pik3cd,* and *Fcgr1*  **Dendritic cell 2** – *Prkcd, Cfl1, Vasp, Mapk3,* and *Scin*  **Dermal Dendritic cells** – *Fcgr4,* and *Fcgr1* |
| Neutrophil extracellular trap formation | **Neutrophils** - *Fpr3, Rela, Pik3cb, Fpr1, C5ar1, Rac1, Casp4, H3f3a, Fcgr3, Syk, Tlr4, Map2k1, Pik3r1, Tlr2, C3, Ncf1, Fpr2, Pik3ca,* and *Cybb*  **Inflammatory Monocytes** - *Pik3cb, Fpr1, Tlr2,* and *Fpr2*  **Macrophage** – *Plcb1, Pik3cb, Hdac5, Hat1, Hdac1, Rac2, Mapk1, Src, Vdac3, Mapk3, Map2k1, Hdac4, Clcn3, Pik3r1, Tlr2, C3,* and *Fpr2*  **Resident Macrophage** – *Plcb2, Fpr1, Fcgr4, C3,* and *Itgal*  **Dendritic cell 1** – *Selplg, Gsdmd, Fcgr3, Tlr2, Fcgr4, Rac2, Fpr2, Cybb, Pik3cd,* and *Fcgr1*  **Dendritic cell 2** – *Casp4, Cybb, H3f3b,* and *Mapk3*  **Dermal Dendritic cells** – *Fpr3, Fpr1, Fcgr4, C3, Fpr2*, and *Fcgr1* |
| Toll-like receptor signaling pathway | **Neutrophils** - *Il1b, Casp8, Spp1, Jun, Nfkbia, Traf6, Tnf, Map2k1, Map3k8, Cd14, Cxcl10, Ccl3, Rela, Pik3cb, Rac1, Stat2, Stat1, Tlr6, Tlr4, Ccl4, Tbk1, Ikbke, Pik3r1, Tlr2, Irf5* and *Pik3ca*  **Inflammatory Monocytes** - *Ccl3, Il1b, Pik3cb, Spp1, Tlr2, Cd40, Ifnar1, Cxcl9, Stat2, Tlr6, Ccl5,* and *Ccl4*  **Macrophage** – *Jak1, Ccl3, Il1b, Pik3cb, Spp1, Traf6, Mapk1, Irf9, Mapk3, Map2k1, Pik3r1, Tlr2, Chuk, Ctsk,* and *Cd14*  **Resident Macrophage** – *Spp1* and *Cxcl9*  **Dendritic cell 1** – *Il1b, Irf7, Ccl3, Tlr2, Cd40, Pik3cd, Stat1* and *Ccl5*  **Dendritic cell 2** – *Il1b, Ccl3, Cd40, Cxcl9, Il12b, Mapk3* and *Ccl4*  **Dermal Dendritic cells** – *Tlr5,* *Spp1* and *Cxcl9* |
| C-type lectin receptor signaling pathway | **Neutrophils** - *Il1b, Casp8, Prkcd, Bcl3, Mdm2, Mapkapk2, Jun, Nfkbia, Tnf, Clec4e, Itpr2, Relb, Malt1, Irf1, Rela, Pik3cb, Clec4d, Stat2, Stat1, Syk, Ikbke, Pik3r1, Clec4n, Fcer1g, Pik3ca,* and *Nlrp3*  **Inflammatory Monocytes** - *Il1b, Ptgs2, Irf1, Pik3cb, Clec4e,* and *Stat2*  **Macrophage** – *Ptpn11, Il1b, Ptgs2, Malt1, Pik3cb, Kras, Clec4d, Mapk1, Src, Irf9, Mapk3, Pik3r1, Clec4n, Plk3, Chuk, Egr2, Nfatc1,* and *Itpr2*  **Dendritic cell 1** – *Cd209a, Il1b, Ptgs2, Malt1, Ccl17, Clec4n, Clec4e, Pik3cd, Stat1* and *Nlrp3*  **Dendritic cell 2** – *Il1b, Ptgs2, Prkcd, Clec4d, Ccl17, Il12b,* and *Mapk3*  **Dermal Dendritic cells** – *Calml4*, and *Ccl17* |
| NOD-like receptor signaling pathway | **Neutrophils** - *Il1b, Nfkbib, Casp8, Prkcd, Cxcl2, Il18, Aim2, Jun, Nfkbia, Rnasel, Traf6, Tnf, Cxcl3, Gbp7, Irgm2, Gbp5, Nampt, Irgm1, Nod1, Itpr2, Mcu, Ctsb, Rela, Ywhae, Casp4, Tank, Txn1, Stat2, Erbin, Stat1, Atg16l1, Tlr4, Tbk1, Ikbke, Hsp90aa1, Mefv, Gabarap, Tnfaip3, Ifi204, Bcl2l1, Cybb, Gbp3, Birc3, Gbp2, Nlrp3,* and *Xiap*  **Inflammatory Monocytes** - *Il1b, Cxcl2, Gbp5, Nampt, Ifnar1, Stat2, Gbp7, Cxcl3, Ccl5, Gbp2,* and *Irgm1*  **Macrophage** – *Jak1, Plcb1, Il1b, Map1lc3b, Cxcl2, Dnm1l, Traf6, Mapk1, Atg16l1, Irf9, Cxcl3, Vdac3, Mapk3, Gabarapl1, Cxcl1, Txnip, Atg5, Chuk, Birc2, Pkn1, Antxr2, Itpr2, Mcu,* and *P2rx7*  **Resident Macrophage** – *Plcb2* and *Trpm2*  **Dendritic cell 1** – *Il1b, Irf7, Gbp5, Gsdmd, Cybb, Stat1, Ccl5, Gbp2, Irgm1,* and *Nlrp3*  **Dendritic cell 2** – *Il1b, Prkcd, Gbp5, Cxcl2, Nampt, Gabarap, Casp4, Txn1, Sugt1, Cybb, Gbp2,* and *Mapk3* |
| HIF-1 signaling pathway | **Neutrophils** - *Hk3, Pgk1, Egln3, Map2k1, Pfkfb3, Eno1, Cdkn1a, Hmox1, Eif4e, Gapdh, Pfkl, Hif1a, Rela, Nos2, Pik3cb, Hk2, Igf1r, Hk1, Stat3, Slc2a1, Tlr4, Ldha, Pik3r1, Pfkp, Pik3ca, Aldoa,* and *Cybb*  **Inflammatory Monocytes** - *Hk3, Vegfa, Hif1a, Pik3cb, Nos2, Serpine1, Hk2, Igf1r, Hk1, Slc2a1,* and *Egln3*  **Macrophage** – *Cul2, Hk3, Egln1, Vegfa, Pgk1, Ltbr, Ifngr2, Mapk1, Egln3, Mapk3, Map2k1, Pfkfb3, Eno1, Cdkn1a, Eif4e, Gapdh, Arnt, Pfkl, Hif1a, Nos2, Pik3cb, Hk2, Igf1r, Hk1, Slc2a1, Ldha, Pik3r1, Trf, Aldoa,* and *Pdhb*  **Resident Macrophage** – *Nos2, Slc2a1* and *Egln3*  **Dendritic cell 1** – *Vegfa, Hif1a, Nos2, Pfkfb3, Cdkn1a, Cybb, Pik3cd, Slc2a1,* and *Egln3*  **Dendritic cell 2** – *Vegfa, Hif1a, Ldha, Hk2, Eno1, Gapdh, Cybb,* and *Mapk3*  **Dermal Dendritic cells** – *Nos2,* and *Egln3* |
| TNF signaling pathway | **Neutrophils** - *Il1b, Casp8, Bcl3, Cxcl2, Junb, Jun, Nfkbia, Tnf, Cxcl3, Map2k1, Csf1, Map3k8, Cxcl10, Cflar, Irf1, Rela, Pik3cb, Icam1, Tnfrsf1b, Atf2, Traf1, Atf4, Socs3, Pik3r1, Tnfaip3, Cebpb, Ifi47, Creb5, Pik3ca, Birc3,* and *Xiap*  **Inflammatory Monocytes** - *Il1b, Ptgs2, Irf1, Pik3cb, Csf1, Cxcl2, Il15, Ifi47, Cxcl3,* and *Ccl5*  **Macrophage** – *Mmp14, Itch, Il1b, Ptgs2, Pik3cb, Cxcl2, Dnm1l, Atf2, Mapk1, Rps6ka5, Cxcl3, Mapk3, Cxcl1, Map2k1, Socs3, Pik3r1, Chuk, Birc2,* and *Jag1*  **Resident Macrophage** – *Mmp14* and *Vcam1*  **Dendritic cell 1** – *Il1b, Ccl5, Ptgs2, Ifi47,* and *Pik3cd*  **Dendritic cell 2** – *Il1b, Ptgs2, Socs3, Cxcl2, Creb5,* and *Mapk3* |
| NF-kappa B signaling pathway | **Neutrophils** - *Il1b, Cxcl2, Ltb, Nfkbia, Traf6, Tnf, Cxcl3, Ube2i, Cd14, Cflar, Relb, Malt1, Rela, Gadd45b, Icam1, Bcl2a1a, Gadd45a, Lyn, Tlr4, Traf1, Ccl4, Syk, Tnfaip3, Bcl2a1b, Bcl2l1, Bcl2a1d, Birc3,* and *Xiap*  **Inflammatory Monocytes** - *Il1b, Ptgs2, Cxcl2, Cd40, Cxcl3*, and *Ccl4*  **Macrophage** – *Il1b, Ptgs2, Malt1, Cxcl2, Ltbr, Gadd45a, Traf6, Cxcl3, Btk, Cxcl1, Chuk, Cd14,* and *Birc2*  **Resident Macrophage** – *Vcam1, Ltb,* and *Bcl2a1a*  **Dendritic cell 1** – *Il1b, Ptgs2, Malt1, Card11, Cd40,* and *Blnk*  **Dendritic cell 2** – *Il1b, Ptgs2, Cxcl2, Cd40* and *Ccl4* |
| Th1 and Th2 cell differentiation | **Neutrophils** - *Stat6, Nfkbib, Rbpj, Rela, Nfkbie, Il2rg, Jun, Jak2, Nfkbia, Il4ra, Stat1, H2-Aa,* and *H2-Ab1*  **Inflammatory Monocytes** – *Rbpj, H2-Eb1,* and *Il4ra*  **Macrophage** – *Jak1, Ifngr2, Il4ra, Chuk, Mapk1, Notch1, Nfatc1, Mapk3,* and *Jag1*  **Resident Macrophage** – *Il2ra, Il12rb2, Runx3, Cd3d,* and *Cd3e*  **Dendritic cell 1** – *Il2rg, Il12rb2, H2-DMb2,* and *Stat1*  **Dendritic cell 2** – *Il12rb2, H2-Eb1, H2-DMb2, Runx3, H2-Aa, H2-Ab1, Il12b, H2-DMa* and *Mapk3* |
| Th17 cell differentiation | **Neutrophils** – *Rara, Il1b, Nfkbib, Stat6, Rela, Hif1a, Nfkbie, Il2rg, Jun, Jak2, Runx1, Nfkbia, Il4ra, Stat3, Tgfb1, Stat1, Tgfbr1, Il1rap, Hsp90aa1, H2-Aa, H2-Ab1* and *Smad3*  **Inflammatory Monocytes** - *Il1b, Hif1a, H2-Eb1, Il4ra,* and *Il1rap*  **Macrophage** – *Il1b, Jak1, Il21r, Hif1a, Ifngr2, Il4ra, Chuk, Mapk1, Nfatc1, Mapk3,* and *Il1rap*  **Resident Macrophage** – *Il21r, Il2ra, Cd3d,* and *Cd3e*  **Dendritic cell 1** – *Il1b, Il21r, Hif1a, Il2rg, H2-DMb2,* and *Stat1*  **Dendritic cell 2** – *Il1b, Hif1a, H2-Eb1, H2-DMb2, H2-Aa, H2-Ab1, H2-DMa, Mapk3* and *Ahr*  **Dermal Dendritic cells** – *Il21r* |
| IL-17 signaling pathway | **Neutrophils** - *Il1b, S100a9, Rela, Casp8, Cxcl2, Jun, Nfkbia, Tnf, Traf6, Hsp90b1, Cxcl3, Tbk1, Ikbke, Lcn2, Hsp90aa1, Tnfaip3, Mapk6, Cebpb, Srsf1, Jund, Cxcl10,* and *Usp25*  **Inflammatory Monocytes** - *Il1b, Ptgs2, S100a8, Cxcl2,* and *Cxcl3*  **Macrophage** – *Il1b, Ptgs2, S100a9, Cxcl1, S100a8, Cxcl2, Srsf1, Chuk, Traf6, Mapk1, Cxcl3,* and *Mapk3*  **Resident Macrophage** – *Lcn2, S100a9,* and *S100a8*  **Dendritic cell 1** – *Il1b, Ptgs2, S100a9, S100a8,* and *Ccl17*  **Dendritic cell 2** – *Il1b, Ptgs2, S100a9, S100a8, Cxcl2, Ccl17,* and *Mapk3*  **Dermal Dendritic cells** – *Lcn2* and *Ccl17* |
| Chemokine signaling pathway | **Neutrophils** - *Nfkbib, Prkcd, Hck, Cxcl2, Pxn, Jak2, Nfkbia, Fgr, Cxcl3, Map2k1, Rap1a, Ncf1, Ptk2b, Cxcl10, Gnai3, Ccl3, Rela, Arrb1, Pik3cb, Rac1, Gnaq, Prex1, Stat3, Stat2, Lyn, Foxo3, Stat1, Ccl6, Vav1, Ccl4, Elmo1, Ccr1, Pik3r1, Vav3, Gsk3a, Gnb1, Pik3ca, Cdc42,* and *Rock2*  **Inflammatory Monocytes** - *Ccl3, Cxcl16, Pik3cb, Cxcl2, Stat2, Cxcl3, Ccl5, Ccl4, Pik3r6, Ccr9, Cxcl9, Ccr5,* and *Arrb2*  **Macrophage** – *Plcb1, Cxcl2, Pxn, Kras, Was, Mapk1, Src, Cxcl3, Mapk3, Map2k1, Chuk, Gngt2, Ccl3, Cxcl16, Arrb1, Pik3cb, Sos1, Rac2, Braf, Foxo3, Pik3r6, Ccr1, Cxcl1, Pik3r1, Prkacb,* and *Gng10*  **Resident Macrophage** – *Plcb2, Cxcl13, Cxcl9, Gngt2,* and *Fgr*  **Dendritic cell 1** – *Ccl3, Ccr1, Ccl17, Rac2, Ccr5, Pik3cd, Stat1, Fgr, Cxcr4, Ccl5,* and *Ccr7*  **Dendritic cell 2** – *Ccl3, Prkcd, Cxcl2, Gnb2, Ccl17, Foxo3, Ccl8, Fgr, Ccl4, Mapk3, Pik3r6, Cxcl9, Gngt2,* and *Grk5*  **Dermal Dendritic cells** – *Ccl17, Cxcl9, Gngt2,* and *Cxcr2* |
| Cytokine-cytokine receptor interaction | **Neutrophils** - *Il1b, Il1a, Cxcl2, Il18, Ltb, Il10rb, Il4ra, Tnf, Tgfb1, Cxcl3, Csf1, Cxcl10, Ccl3, Il13ra1, Il2rg, Inhba, Il15ra, Tnfrsf1b, Ccl6, Tgfbr1, Il1rn, Ccl4, Il1rap, Ccr1, Csf2rb,* and *Csf2rb2*  **Inflammatory Monocytes** - *Ccl3, Il1b, Il7r, Il1a, Cxcl16, Cxcl2, Inhba, Il15, Il4ra, Cxcl3, Ccl5, Il1rn, Ccl4, Il1rap, Csf1, Ccr9, Cd40, Ifnar1, Cxcl9, Ccr5, Csf2rb,* and *Csf2rb2*  **Macrophage** – *Ccl3, Il1b, Il13ra1, Il7r, Cxcl16, Cxcl2, Inhba, Ltbr, Ifngr2, Il1r2, Il4ra, Cxcl3, Il1rn, Il1rap, Tnfrsf9, Il21r, Ccr1, Cxcl1, Tgfb3, Csf2rb,* and *Csf2rb2*  **Resident Macrophage** – *Il21r, Il18rap, Il12rb2, Il2ra, Ltb, Inhba, Cxcl13, Il1r2,* and *Cxcl9*  **Dendritic cell 1** – *Ccl3, Il1b, Il7r, Il2rg, Il12rb2, Osm, Inhba, Ccl17, Ccl5, Il1rn, Il21r, Ccr1, Cd40, Tnfsf4, Ccr5, Cxcr4,* and *Ccr7*  **Dendritic cell 2** – *Ccl3, Il1b, Il12rb2, Cxcl2, Acvr2a, Il1r2, Ccl17, Ccl8, Il12b, Il1rn, Ccl4, Cd40,* and *Cxcl9*  **Dermal Dendritic cells** – *Il21r, Osm, Inhba, Ccl17, Cxcl9, Csf3r,* and *Cxcr2* |
| JAK-STAT signaling pathway | **Neutrophils** - *Stat6, Jak2, Il10rb, Il4ra, Cdkn1a, Pim1, Il13ra1, Il2rg, Pik3cb, Il15ra, Ptpn2, Mcl1, Stat3, Stat2, Stat1, Ptpn6, Stam2, Socs3, Socs1, Pik3r1, Csf2rb, Bcl2l1, Pik3ca, Cish,* and *Csf2rb2*  **Inflammatory Monocytes** - *Il7r, Pik3cb, Socs1, Il15, Ifnar1, Il4ra, Csf2rb, Stat2,* and *Csf2rb2*  **Macrophage** – *Jak1, Ptpn11, Il13ra1, Il7r, Pik3cb, Sos1, Ifngr2, Ptpn2, Il4ra, Irf9, Stam2, Il21r, Pias1, Socs3, Pik3r1, Cdkn1a, Csf2rb, Socs4, Cish,* and *Csf2rb2*  **Resident Macrophage** – *Il21r, Il2ra,* and *Il12rb2*  **Dendritic cell 1** – *Il21r, Il7r, Il2rg, Il12rb2, Osm, Cdkn1a, Pik3cd, Stat1,* and *Cish*  **Dendritic cell 2** – *Socs3, Il12rb2,* and *Il12b*  **Dermal Dendritic cells** – *Il21r, Osm,* and *Csf3r* |
| Arginine biosynthesis | **Neutrophils** - *Nos2*  **Inflammatory Monocytes** - *Got1, Nos2,* and *Arg1*  **Macrophage** – *Got1, Nos2, Arg2, Arg1,* and *Ass1*  **Resident Macrophage** – *Nos2, Arg2, Arg1*, and *Ass1*  **Dendritic cell 1** – *Nos2* and *Arg1*  **Dendritic cell 2** – Got1, Arg2, Arg1, and Ass1  **Dermal Dendritic cells** – *Nos2* and *Arg1* |
| Antigen processing and presentation | **Neutrophils** - *Ctss, Hspa8, H2-T23, Psme2, Canx, B2m, Tnf, Psme1, Hspa1a, H2-Q10, H2-K1, H2-Q6, Ctsb, Tapbp, Tap2, Tap1, Hspa5, Hsp90aa1, H2-Q7, H2-T22, Cd74, Hspa1b, H2-Aa, H2-Ab1,* and *H2-D1*  **Inflammatory Monocytes** - *Tap1,* and *H2-Eb1*  **Macrophage** – *Ctss, Hspa1a, H2-Q6, Ifi30, Nfya,* and *Ctsl*  **Dendritic cell 1** – *Cd8b1, Tap1, Ifi30, H2-DMb2,* and *Cd8a*  **Dendritic cell 2** – *Hspa1a, H2-Q6, H2-Eb1, Tap1, Psme2, H2-DMb2, H2-Q7, Ciita, H2-Aa, Cd74, H2-Ab1,* and *H2-DMa* |
| Complement and coagulation cascades | **Neutrophils** - *C5ar1, Plaur, F3, C3,* and *F10*  **Inflammatory Monocytes** - *C1qb, Serpine1, F3, Cfb, F13a1, F10,* and *C3ar1*  **Macrophage** – *Procr, Plaur, Cfb, C3, F10, Thbd,* and *Serpinb2*  **Resident Macrophage** – [*F7*](http://www.ncbi.nlm.nih.gov/entrez/query.fcgi?db=gene&cmd=Retrieve&dopt=Graphics&list_uids=F7)*,* [*C3*](http://www.ncbi.nlm.nih.gov/entrez/query.fcgi?db=gene&cmd=Retrieve&dopt=Graphics&list_uids=C3) and [*F10*](http://www.ncbi.nlm.nih.gov/entrez/query.fcgi?db=gene&cmd=Retrieve&dopt=Graphics&list_uids=F10)  **Dendritic cell 1** – *F10*  **Dendritic cell 2** – *Procr*  **Dermal Dendritic cells** – [*F7*](http://www.ncbi.nlm.nih.gov/entrez/query.fcgi?db=gene&cmd=Retrieve&dopt=Graphics&list_uids=F7)*,* [*C3*](http://www.ncbi.nlm.nih.gov/entrez/query.fcgi?db=gene&cmd=Retrieve&dopt=Graphics&list_uids=C3) and *Cfb* |
| PI3K-Akt signaling pathway | **Neutrophils** - *Mdm2, Spp1, Itgav, Jak2, Il4ra, Ywhaz, Map2k1, Csf1, Ppp2r2d, Cdkn1a, Eif4e, Itgb1, Pik3ap1, Lamb3, Rela, Il2rg, Pik3cb, Ddit4, Ywhae, Rac1, Igf1r, Pten, Mcl1, Ppp2ca, Crtc2, Atf2, Foxo3, Hsp90b1, Tlr4, Syk, Atf4, Hsp90aa1, Pik3r1, Ppp2r1a, Tlr2, Gnb1, Creb5, Bcl2l1,* and *Pik3ca*  **Inflammatory Monocytes** - *Vegfa, Il7r, Pik3cb, Spp1, Met, Igf1r, Il4ra, Pik3r6, Csf1, Gys1, Tlr2, Ifnar1,* and *Lpar1*  **Macrophage** – *Jak1, Vegfa, Il7r, Pgf, Spp1, Kras, Itgav, Il4ra, Thbs1, Lamc1, Mapk1, Ppp2r5e, Mapk3, Map2k1, Gys1, Cdkn1a, Sgk1, Chuk, Gngt2, Eif4e, Pkn1, Ppp2r2a, Itga1, Pik3cb, Ddit4, Sos1, Met, Itgb7, Igf1r, Pten, Atf2, Foxo3, Pik3r6, Pik3r1, Tlr2, Bcl2l11, Pdpk1, Gng10,* and *Cdk4*  **Resident Macrophage** – *Pgf, Il2ra, Spp1, Hgf, Gngt2,* and *Thbs1*  **Dendritic cell 1** – *Vegfa, Il7r, Il2rg, Ddit4, Pgf, Gys1, Osm, Tlr2, Areg, Cdkn1a,* and *Pik3cd*  **Dendritic cell 2** – *Vegfa, Pik3r6, Ddit4, Ywhag, Gnb2, Creb5, Gngt2, Bcl2l11, Foxo3, Ywhaq, Kit,* and *Mapk3*  **Dermal Dendritic cells** – *Gys1, Osm, Spp1, Gngt2,* and *Csf3r* |
