# Supplementary material for: Single-cell transcriptomics unveils skin cell specific antifungal immune responses and IL-1Ra- IL-1R immune evasion strategies of emerging fungal pathogen Candida auris
Source: PLoS Pathog. 2024 Nov 13;20(11):e1012699. doi: 10.1371/journal.ppat.1012699 (PMC11588283; doi:10.1371/journal.ppat.1012699)
Supplement: S4 Table — (DOCX) [file ppat.1012699.s011.docx]

**Table S4:** The DEGs of lymphoid subsets enriched in the KEGG pathways upon *C. auris* murine skin infection.

| **KEGG Pathways** | **DEGs enriched in the pathway** |
| --- | --- |
| Th17 cell differentiation | **CD4+ Th Cells** - *Il23r, Stat5b, Hif1a, Stat5a, Il2ra, Il22, Il1r1, Nfkbia, Il17a, Lck, Cd4, Ifng, Cd247,* and *Cd3d*  **CD8+ Cells** – *Nfkbia*  **γδ+ T cells** – *Hif1a, Il22,* and *Ifng* |
| Th1 and Th2 cell differentiation | **CD4+ Th Cells** – *Lck, Stat5b, Stat5a, Cd4, Il2ra, Nfkbia, Cd247, Ifng* and *Cd3d*  **CD8+ Cells** – *Nfkbia*  **γδ+ T cells** – *Il13, Il12rb2,* and *Ifng* |
| IL-17 signaling pathway | **CD4+ Th Cells** – *Nfkbia, Csf2, Ifng, Il17a*, and *Cxcl3*  **CD8+ Cells** – *S100a9, S100a8, Cxcl2* and *Nfkbia*  **γδ+ T cells** – *Ptgs2, Il13, Csf2,* and *Ifng* |
| TNF signaling pathway | **CD4+ Th Cells** - *Csf1, Nfkbia, Csf2, Cxcl3* and *Cflar*  **Tregs** - *Map2k1*  **CD8+ Cells** – *Cxcl2* and *Nfkbia*  **γδ+ T cells** – *Ptgs2, Csf2, Ccl5* and *Cflar* |
| T cell receptor signaling pathway | **CD4+ Th Cells** – *Lck, Cd4, Nfkbia, Ctla4, Csf2, Nck2, Cd247, Ifng,* and *Cd3d*  **Tregs** - *Map2k1*  **CD8+ Cells** – *Icos,* and *Nfkbia*  **γδ+ T cells** – *Ctla4, Csf2,* and *Ifng* |
| B cell receptor signaling pathway | **CD4+ Th Cells** - *Lilrb4a, Ifitm1, Nfkbia,* and *Pik3ap1*  **Tregs** - *Map2k1* and *Prkcb*  **CD8+ Cells** – *Nfkbia* |
| Chemokine signaling pathway | **CD4+ Th Cells** - *Stat5b, Ccr1, Plcb4, Nfkbia, Gnaq,* and *Cxcl3*  **Tregs** – *Prkcb, Map2k1,* and *Ccl4*  **CD8+ Cells** – *Cxcl2, Nfkbia,* and *Ccl4*  **γδ+ T cells** – *Ccr5* and *Ccl5* |
| Cytokine-cytokine receptor interaction | **CD4+ Th Cells** - *Il23r, Stat5b, Hif1a, Stat5a, Il2ra, Il22, Il1r1, Nfkbia, Il17a, Lck, Cd4, Ifng, Cd247,* and *Cd3d*  **Tregs** - *Il1r2* and *Ccl4*  **CD8+ Cells** –*Cxcl2, Il1rn,* and *Ccl4*  **γδ+ T cells** – *Il13, Il18rap, Il12rb2, Il22, Il1r2, Ccr5, Csf2, Ifng,* and *Ccl5* |
| JAK-STAT signaling pathway | **CD4+ Th Cells** - *Il23r, Stat5b, Stat5a, Il2ra, Il22, Csf2, Pim1,* and *Ifng*  **γδ+ T cells** – *Il13, Il12rb2, Il22, Pdgfb, Csf2,* and *Ifng* |
| NF-kappa B signaling pathway | **CD4+ Th Cells** – *Lck, Ltb, Il1r1, Nfkbia, Cxcl3,* and *Cflar*  **Tregs** - *Prkcb* and *Ccl4*  **CD8+ Cells** – *Cxcl2, Ccl4* and *Nfkbia*  **γδ+ T cells** – *Cflar* and *Ptgs2* |
| HIF-1 signaling pathway | **CD4+ Th Cells** - *Pfkl, Egln1, Hif1a, Ldha, Pgk1, Pfkp, Eno1,* and *Ifng*  **Tregs** - *Egln1, Prkcb, Map2k1, Hk2* and *Prkca*  **γδ+ T cells** – *Hif1a, Eno1,* and *Ifng* |
| VEGF signaling pathway | **CD4+ Th Cells** - *Mapkapk2*  **Tregs** – *Prkcb, Map2k1* and *Prkca*  **γδ+ T cells** – *Ptgs2* and *Sh2d2a* |
| Wnt signaling pathway | **CD4+ Th Cells** - *Plcb4, Lef1,* and *Ccn4*  **Tregs** – *Prkcb, Prkca, Tbl1x,* and *Lef1* |
| Rap1 signaling pathway | **CD4+ Th Cells** - *Csf1, Plcb4, Gnaq, Rapgef1,* and *Itgal*  **Tregs** – *Prkcb, Map2k1,* and *Prkca*  **CD8+ Cells** – *Thbs1*  **γδ+ T cells** – *Pdgfb*  **NK Cells** – *Evl* |
| Fc epsilon RI signaling pathway | **CD4+ Th Cells** – *Csf2*  **Tregs** - *Map2k1,* and *Prkca*  **γδ+ T cells** – *Csf2* and *Il13* |
| Fc gamma R-mediated phagocytosis | **CD4+ Th Cells** - *Myo10,* and *Actr3*  **Tregs** – *Prkcb, Map2k1,* and *Prkca*  **γδ+ T cells** – *Myo10* |
| Natural killer cell mediated cytotoxicity | **CD4+ Th Cells** – *Lck, Klrc1, Csf2, Itgal, Cd247,* and *Ifng*  **Tregs** – *Prkcb, Map2k1, Gzmb,* and *Prkca*  **γδ+ T cells** – *Klrc2, Klrc1, Csf2,* and *Ifng* |
