# Supplementary material for: Single-cell transcriptomics unveils skin cell specific antifungal immune responses and IL-1Ra- IL-1R immune evasion strategies of emerging fungal pathogen Candida auris
Source: PLoS Pathog. 2024 Nov 13;20(11):e1012699. doi: 10.1371/journal.ppat.1012699 (PMC11588283; doi:10.1371/journal.ppat.1012699)
Supplement: S5 Table — (DOCX) [file ppat.1012699.s012.docx]

**Table S5:** The DEGs of fibroblast subsets enriched in the KEGG pathways upon *C. auris* murine skin infection.

| **KEGG Pathways** | **DEGs enriched in the pathway** |
| --- | --- |
| HIF-1 signaling pathway | **Fibroblast 1** - *Nos2, Serpine1* and *Timp1*  **Fibroblast 3** – *Igf1, Pfkl, Nos2, Serpine1, Elob, Eno1, Eif4ebp1, Timp1, Slc2a1* and *Angpt1* |
| PI3K-Akt signaling pathway | **Fibroblast 1** - *Ddit4, Pgf, Ereg, Col6a5, Tnc, Pik3ap1,* and *Csf3*  **Fibroblast 2**- *Csf3r*  **Fibroblast 3** – *Igf1, Ddit4, Pgf, Spp1, Itga11, Fgf23, Thbs4, Thbs3, Tnc, Creb3l3, Col6a2, Gys1, Ereg, Eif4ebp1, Tnn, Angpt1,* and *Csf3* |
| ECM-receptor interaction | **Fibroblast 1** - *Col6a5* and *Tnc*  **Fibroblast 3** – *Col6a2, Spp1, Itga11, Thbs4, Thbs3, Tnn,* and *Tnc* |
| IL-17 signaling pathway | **Fibroblast 1** - *Lcn2, S100a9, S100a8, Mmp13, Cxcl3,* and *Csf3*  **Fibroblast 2**- *S100a9, S100a8, Cxcl2,* and *Cxcl3*  **Fibroblast 3** – *Lcn2, S100a9, Cxcl5, Mmp13,* and *Csf3*  **Fibroblast 4** – *S100a9* |
| NF-kappa B signaling pathway | **Fibroblast 1** - *Cxcl3*  **Fibroblast 2**- *Cxcl3, Btk* and *Cxcl2*  **Fibroblast 3** – *Lbp* and *Cxcl12* |
| Cytokine-cytokine receptor interaction | **Fibroblast 1** - *Il13ra2, Il33, Cxcl9, Tnfsf8, Cxcl3,* and *Csf3*  **Fibroblast 2**- *Cxcl2, Csf3r,* and *Cxcl3*  **Fibroblast 3** – *Cxcl5, Cxcl14, Il33, Il11ra1, Bmpr1b, Tnfsf8, Il18r1, Cxcl12* and *Csf3* |
| TNF signaling pathway | **Fibroblast 1** - *Cxcl3*  **Fibroblast 2**- *Cxcl3,* and *Cxcl2*  **Fibroblast 3** – *Cxcl5, Creb3l3* and *Il18r1* |
| Chemokine signaling pathway | **Fibroblast 1** - *Cxcl9* and *Cxcl3*  **Fibroblast 2**- *Cxcl3* and *Cxcl2*  **Fibroblast 3** – *Cxcl5, Cxcl14* and *Cxcl12* |
| NOD-like receptor signaling pathway | **Fibroblast 1** - *Cxcl3*  **Fibroblast 2**- *Cxcl3* and *Cxcl2*  **Fibroblast 3** – *Irf7* |
| Complement and coagulation cascades | **Fibroblast 1** - *Kng2, Serpine1, Cfb, Cfi,* and *Serpinb2*  **Fibroblast 3** – *Bdkrb2, Serpine1, Cfb, C3,* and *Serpinb2* |
