# Supplementary material for: Single-cell transcriptomics unveils skin cell specific antifungal immune responses and IL-1Ra- IL-1R immune evasion strategies of emerging fungal pathogen Candida auris
Source: PLoS Pathog. 2024 Nov 13;20(11):e1012699. doi: 10.1371/journal.ppat.1012699 (PMC11588283; doi:10.1371/journal.ppat.1012699)
Supplement: S6 Table — (DOCX) [file ppat.1012699.s013.docx]

**Table S6:** The list of antibodies, ELISA kit, and recombinant proteins used in the study.

| **Reagent or Resource** | **Source** | **Identifier** |
| --- | --- | --- |
| **Antibodies** | | |
| Anti-mouse CD11c, APC (N418) | Biolegend | Cat# 117309; RRID: AB_313779 |
| Anti-mouse Ly6C, Pacific Blue (HK1.4) | Biolegend | Cat# 128014; RRID: AB_1732079 |
| Anti-mouse CD11b, PE (M1/70) | Biolegend | Cat# 101208; RRID: AB_312791 |
| Anti-mouse Ly6G, PE/Cyanine7 (1A8) | Biolegend | Cat# 127618; RRID: AB_1877261 |
| Anti-mouse Ly6G, APC (S19018G) | Biolegend | Cat#164506; RRID: AB_2927993 |
| Anti-mouse CD45, FITC (30-F11) | Biolegend | Cat# 103108; RRID: AB_312973 |
| Anti-mouse CD64, PE/Dazzle 594 (X54-5/7.1) | Biolegend | Cat# 139320; RRID: AB_2566559 |
| Anti-mouse MHC II, Alexa Fluor 700 (M5/114.15.2) | eBioscience | Cat# 56532182; RRID: AB_494009 |
| Anti-mouse TCR γ/δ, BV421 (GL3) | Biolegend | Cat# 118120; RRID: AB_2562566 |
| Anti-mouse CD4, PerCP/Cyanine5.5 (GK1.5) | Biolegend | Cat# 100434; RRID: AB_893324 |
| Anti-mouse CD8b, PE (H35-17.2) | BD Biosciences | Cat# 550798; RRID: AB_393887 |
| Anti-mouse TCR β, PE/Cyanine7 (H57-597) | Biolegend | Cat# 109222; RRID: AB_893625 |
| Anti-mouse IL-17F, Alexa Fluor 488 (9D3.1C8) | Biolegend | Cat# 517006; RRID: AB_10661903 |
| Anti-mouse IL-17A, PE/Dazzle 594 (TC11-18H10.1) | Biolegend | Cat# 506938; RRID: AB_2564321 |
| Anti-mouse IFN-γ, Alexa Fluor 700 (XMG 1.2) | Biolegend | Cat# 505824; RRID: AB_2561300 |
| Anti-mouse CD16/32 (93) | Biolegend | Cat# 101302; RRID: AB_312801 |
| Anti-mouse F4/80, PE/Dazzle 594 (BM8) | Biolegend | Cat# 123145; RRID: AB_2564132 |
| Anti-mouse F4/80, Alexa Fluor 700 (BM8) | Biolegend | Cat# 123129; RRID: AB_2277848 |
| Ultra-LEAF™ Purified anti-mouse Ly-6G Antibody (1A8) | Biolegend | Cat# 127649; RRID: AB_2572001 |
| Ultra-LEAF™ Purified Rat IgG2a, κ Isotype Ctrl Antibody (RTK2758) | Biolegend | Cat# 400565; RRID: AB_11147167 |
| Anti-mouse IL-1Ra monoclonal antibody | This study | Gift from Naofumi Mukaida |
| **ELISA kit** | | |
| Mouse IL-1ra/IL-1F3 DuoSet ELISA | RnD | Cat# DY480 |
| **Recombinant Protein** | | |
| Recombinant Mouse IL-1RA (IL-1RN) | Biolegend | Cat# 769704 |
