# Supplementary material for: Single-cell transcriptomics unveils skin cell specific antifungal immune responses and IL-1Ra- IL-1R immune evasion strategies of emerging fungal pathogen Candida auris
Source: PLoS Pathog. 2024 Nov 13;20(11):e1012699. doi: 10.1371/journal.ppat.1012699 (PMC11588283; doi:10.1371/journal.ppat.1012699)
Supplement: S7 Table — (DOCX) [file ppat.1012699.s014.docx]

**Table S7:** The oligonucleotide sequences used to construct *pmr1* deletion and the confirmation of the desired edit by Sanger sequencing.

| **Description** | Sequence (5’ → 3’) |
| --- | --- |
| B9J08_000837 (*pmr1*) deletion gRNA | TATCGGAAAAGAACCCGTCG |
| Fragment 1 Forward Primer | TGGCGCTCTAGCACATTACC |
| Fragment 1 Reverse Primer | CCTCATGTCGAGCACTCGTCTCGCCGAGGATATGTATTAGGGG |
| Fragment 2 Forward Primer | CGAGACGAGTGCTCGACATGAGGTAGCCCACTTCATGTTGAA |
| Fragment 2 Reverse Primer | CCTCCAGTTTCTACAGAACAGGTGG |
| Colony PCR Forward Primer | CGCTCCGCCATATAACCCAT |
| Colony PCR Reverse Primer | ACGTAACTAGCCTTGACGGG |
| Sanger sequencing confirmation of *pmr1Δ* stain | NNNNNNNNTCTNGTTTGNGGTGCTCGCGCTCCGCCATATAACCCATGAATCCTTCATCGATTCCACCTGGGGTCCCTATA  TCTCCCAAACCACTAGCATCCCTCGCCCACAACCCAACCCCCTAATACATATCCTCGGCGAGACGAGTGCTCGACATGAG  GTAGCCCACTTCATGTTGAACAGCCCCGTCAAGGCTAGTTACGTATATATTATTTAGAGATTTCTGTCTAGAAATCGACA  ATATCATCAAGACTAACAACGCCATTGGCTTCTGGGTCCTCAAGCTTGCGATAAAGCGTGATGGCCACCTGTTCTGNNNA  ANCTGGNNGN |
