# Supplementary material for: Single-cell transcriptomics unveils skin cell specific antifungal immune responses and IL-1Ra- IL-1R immune evasion strategies of emerging fungal pathogen Candida auris
Source: PLoS Pathog. 2024 Nov 13;20(11):e1012699. doi: 10.1371/journal.ppat.1012699 (PMC11588283; doi:10.1371/journal.ppat.1012699)
Supplement: S8 Table — (DOCX) [file ppat.1012699.s015.docx]

**Table S8:** The fungal strains, plasmids and mouse strains used in the study.

| **Fungal Strains** | | | |
| --- | --- | --- | --- |
| **Strains** | **Source** | **Genotype** | |
| *Candida auris* AR0387 | CDC | | Wild type |
| *Candida auris* AR0381 | CDC | | Wild type |
| *Candida auris* AR0383 | CDC | | Wild type |
| *Candida auris* AR0385 | CDC | | Wild type |
| *C. auris* AR0387 *pmr1Δ* | This Study | | *pmr1Δ* |
| *Candida albicans* SC5314 | Gifted from Dr. Andrew Koh | | Wild type |
| **Experimental Models: Organisms/Strains** | | | |
| **Strains** | **Source** | **Reference identifier** | |
| C57BL/6J mice | The Jackson Laboratory | | Cat#000664;  RRID: IMSR_JAX:000664 |
| *IL-1R1^−/−^* C57BL/6J mice | The Jackson Laboratory | | Cat# 003245  RRID: IMSR_JAX:003245 |
| **Plasmids** | **Description** | | **Identifier** |
| pCE35 | CAS9 expression cassette | | #174409 |
| pCE27 | gRNA expression cassette | | #174405 |
