# Supplementary material for: Single-cell transcriptomics unveils skin cell specific antifungal immune responses and IL-1Ra- IL-1R immune evasion strategies of emerging fungal pathogen Candida auris
Source: PLoS Pathog. 2024 Nov 13;20(11):e1012699. doi: 10.1371/journal.ppat.1012699 (PMC11588283; doi:10.1371/journal.ppat.1012699)
Supplement: S9 Table — (DOCX) [file ppat.1012699.s016.docx]

**Table S9:** The list of reagents and software used in this study

| **Chemicals, Peptides, and Recombinant Proteins** | | |
| --- | --- | --- |
| Yeast Extract-Peptone-Dextrose (YPD) | BD Bioscience | Cat # 242810 |
| Recombinant Mouse IL-1RA (IL-1RN) | Biolegend | Cat# 769704 |
| Agar | Fisher Scientific | Cat # BP1423-500 |
| 10× Phosphate Buffer Solution (PBS) | Fisher Scientific | Cat # BP3994 |
| Ampicillin | Cayman Chemicals | Cat # 14417 |
| Streptomycin | MP Biomedicals | Cat # 100556 |
| Bovine Serum Albumin (BSA) | Sigma Aldrich | Cat # A4737 |
| RPMI 1640 Medium (1X) with Glutamine and Phenol Red | ThermoFisher | Cat # 11875093 |
| Fetal Bovine Serum (FBS) | CPS Serum | Cat #: FBS-500HI |
| Percoll | Sigma Aldrich | Cat # P1644 |
| Triton X-100 | MP Biomedicals | Cat # 807423 |
| HyClone RPMI 1640 media with L-glutamine | Cytiva | Cat # SH30027.02 |
| Trypsin-EDTA (0.25%), phenol red | ThermoFisher | Cat # 25200056 |
| Cell Staining Buffer | Biolegend | Cat # 420201 |
| Intracellular Staining Permeabilization Wash Buffer | Biolegend | Cat # 421002 |
| Fixation Buffer | Biolegend | Cat # 420801 |
| Monensin Solution (1,000X) | Biolegend | Cat # 420701 |
| Cell Activation Cocktail (without Brefeldin A) | Biolegend | Cat # 423302 |
| LIVE/DEAD™ Fixable Yellow Dead Cell Stain kit, (405 nm) excitation | Invitrogen, ThermoFisher | Cat # L34959 |
| Liberase TL Research Grade | Sigma Aldrich | Cat # 05401020001 |
| Heparinized capillary tubes | Fisher Scientific | Cat # 22-260950 |
| Deoxyribonuclease I from bovine pancreas | Sigma Aldrich | Cat # DN25-100G |
| Sterile Cell Strainer, 70 µm | Fisherbrand | Cat# 22-363-548 |
| Sterile Cell Strainer, 40 µm | Fisherbrand | Cat# 22-363-547 |
| MACS Smart Strainers | Miltenyi Biotec | Cat# 130-098-458 |
| **Software and Algorithms** | | |
| FlowJo v9 | Tree Star | [https://www.flowjo.com/solutions/flowjo/downloads](https://flowjo.com/solutions/flowjo/downloads) |
| GraphPad Prism 4 | GraphPad Software | [https://www.graphpad.com/scientificsoftware/prism/](https://graphpad.com/scientificsoftware/prism/) |
